# The effect of exercise time and frequency on the development of cam morphology

**DOI:** 10.1186/s12891-025-08603-1

**Published:** 2025-10-01

**Authors:** Mustafa Al-Baldawi, Matthew Pettit, Vikas Khanduja

**Affiliations:** 1 University of Cambridge School of Clinical Medicine, Cambridge, UK; 2 St George’s University, London, UK; 3 Young Adult Hip Service, Department of Trauma and Orthopaedics, Box 37, Hills Road, Addenbrooke’s - Cambridge University Hospitals NHS Foundation Trust, Cambridge, UK

**Keywords:** FAI, Cam morphology, Alpha-angle, Hip, Prevalence, Athletic activity, Sports training, Physical activity levels, Exercise intensity, Scoping review

## Abstract

**Objective:**

Femoroacetabular impingement syndrome (FAIS) with cam morphology is a prevalent cause of intra-articular hip pain in young adults and is widely associated with sporting activity during skeletal immaturity. The exact association between activity levels and cam morphology is not fully understood. This scoping reviews aims to (1) systematically map the available evidence regarding the association between physical activity levels and cam morphology and (2) elucidate the nature of this association, examining whether a threshold effect exists at specific training intensities or durations that could inform preventative guidelines.

**Methods:**

A systematic literature search of Embase, MEDLINE and the Cochrane Library was conducted following the PRISMA-ScR guidelines. Studies investigating the association between activity levels and cam were included. Data was charted and summarised narratively with illustrative figures displaying the odds ratio for cam prevalence and the mean difference in α-angle values between high and low activity groups. Data on training time and cam prevalence were plotted to visualise potential dose-response relationships and threshold effects. Study quality was assessed using the Downs and Black criteria.

**Results:**

The search identified nine studies involving 890 participants (684 males, 206 females). The methodological quality of the included studies ranged from 44 to 95%, with higher percentages indicating better quality. High activity groups showed greater prevalence of cam morphology per individual and per hip compared to low activity groups. α-angle values were also greater in high activity groups. Evidence was heterogeneous in cam prevalence measurement methods, definitions of activity groups and study populations. Inconsistent reporting of exercise time and frequency precluded meaningful characterisation of their association with cam morphology.

**Conclusion:**

This review maps evidence suggesting that cohorts reporting high sporting activity levels (approximately 3–8 training sessions per week or 3.5–12.5 h of weekly training) have greater prevalence of cam morphology and larger α-angle values compared to less active cohorts. Significant knowledge gaps exist in the understanding of the relationship between exercise time and frequency and cam development. Prospective cohort studies with standardised reporting of activity levels and clear stratification of adolescent sporting activity are needed to better characterise this relationship and inform evidence-based guidelines for youth sports participation.

**Clinical trial number:**

Not applicable.

**Supplementary Information:**

The online version contains supplementary material available at 10.1186/s12891-025-08603-1.

## Introduction

Femoroacetabular impingement syndrome (FAIS) is a common cause of intra-articular hip pain in the young adult and has been proposed to be a precursor for the development of idiopathic osteoarthritis of the hip [1–4]. FAIS is characterised by a triad of symptoms, clinical signs and radiographic findings [5]. The underlying hip morphology variants associated with FAIS include pincer, cam, and a mixed type with features of both the former two. Pincer describes an acetabular over-coverage which damages the labrum during repeated linear contact during hip flexion [1]. Conversely, cam morphology describes an abnormal contour of the head-neck junction with reduced head-neck offset. This results in the abutment of the non-spherical femoral head against the acetabulum during hip flexion causing chondro-labral junction abrasion and eventually detachment [1].

Cam FAI morphology (cam) has a male predominance, with an estimated male: female ratio of 4.25:1 [6]. Cam also shows increased prevalence in athletic populations, where it is widely postulated to develop secondary to high levels of sporting activity during skeletal immaturity [7–9]. This is known as the adaptive theory; whereby adaptive changes occur within the physeal plate in response to the stress exerted by high intensity sports.

A systematic review and meta-analysis by Nepple et al. (2015) established that male athletes have a greater prevalence of cam deformity compared to controls [10]. However, this analysis primarily stratified cohorts into athletes vs. controls without examining the magnitude of training volume and frequency. The nature of the relationship between activity levels and cam remains poorly understood. It is unclear whether the physeal changes in response to activity have a threshold of cumulative stress above which adaptations occur or whether a dose-response type relationship exists.

This scoping review builds upon previous work by updating the evidence base and aims to map the available evidence examining the association between training volume and cam, and chart existing research limitations regarding the nature of this association. While previous work has established an association between athletic participation and cam morphology, significant knowledge gaps remain regarding the specific exercise parameters that influence cam development. Key unanswered questions include: (1) if a dose-response relationship exists between training volume and cam development; (2) whether a threshold of cumulative stress exists above which physeal adaptations occur; (3) what combinations of training frequency, duration and intensity pose the greatest risk and (4) whether sport-specific biomechanical demands modify this relationship. Understanding these aspects is essential for developing evidence-based recommendations for youth sports participation that minimise the risk of cam development while promoting physical activity [11].

The purpose of this scoping review, therefore, was to (1) systematically map the available evidence regarding the association between physical activity levels and cam morphology and (2) elucidate the nature of this association, examining whether a threshold effect exists at specific training intensities or durations that could inform preventative guidelines.

## Methods

We performed this scoping review in accordance with the methodological framework outlined by Arksey and O’Malley [12] and advanced by others [13, 14]. The PRISMA-ScR (Preferred Reporting Items for Systematic Reviews and Meta-analyses extension for Scoping Reviews) guidelines [15] and the guidance by the Joanna Briggs Institute [16] were also followed. The protocol for this study was prospectively registered with PROSPERO (CRD42023408796). While initially planned as a systematic review, the methodology was adapted to a scoping review framework given the heterogeneity in how activity levels and cam morphology are measured across studies. This approach allowed mapping the evidence to identify knowledge gaps in the understanding of the specific relationship between exercise parameters and cam development, rather than synthesise findings from homogenous studies.

### Search strategy

**Fig. 1:**
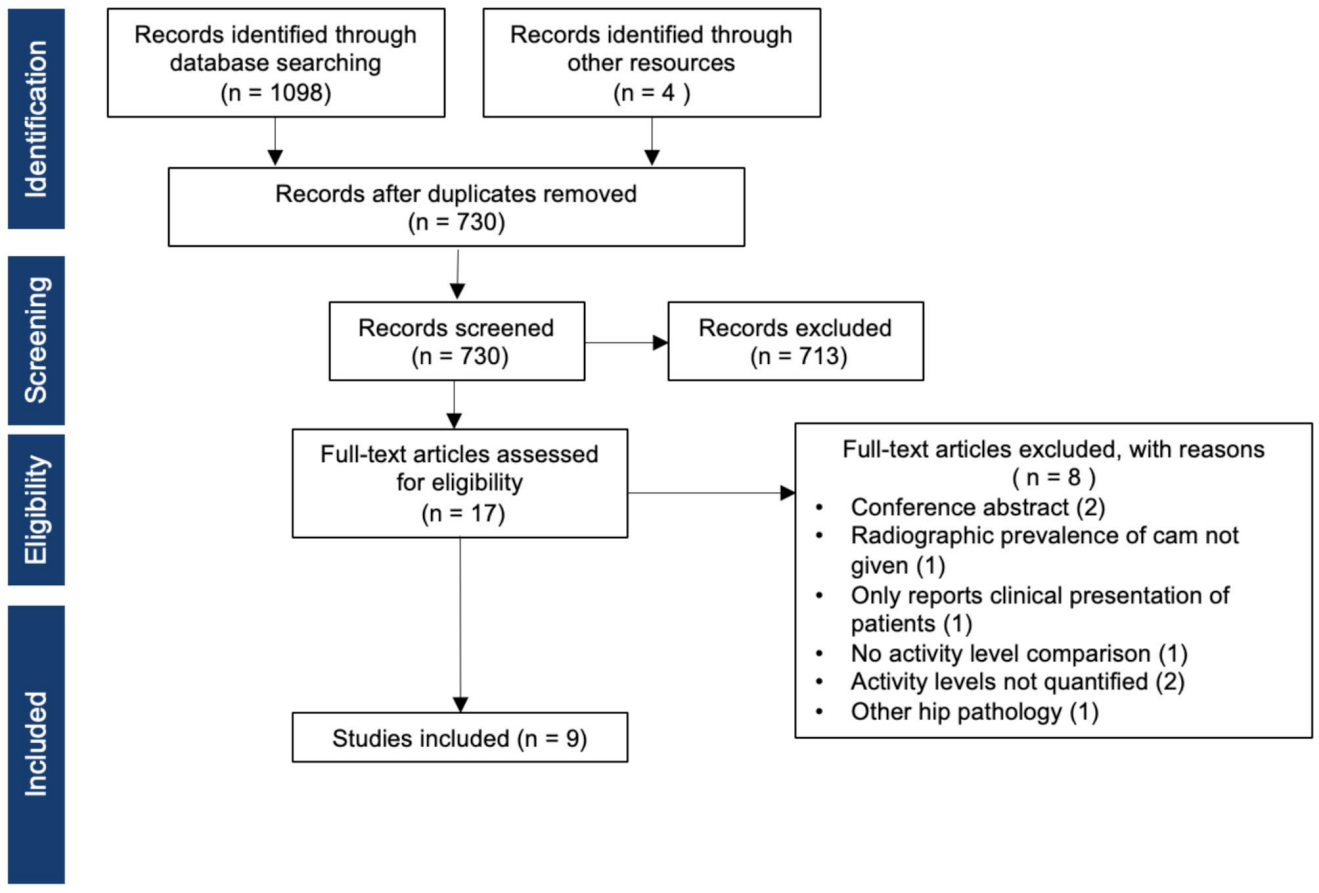
The search processes

The search process is shown in Fig. 1. One reviewer carried out searches across MEDLINE, Embase and Cochrane library databases using key terms of (time OR frequency OR activity level OR intensity) AND (prevalence OR risk) AND (femoroacetabular impingement) with corresponding subheading and free terms. The detailed search strategy is included in supplementary Table I. References were searched to identify further studies.

Two reviewers (M.A., M.P.) independently screened the titles and abstracts. The full texts were then also assessed independently by two authors (M.A., M.P.). This study aimed to map the evidence on the natural relationship between activity levels and cam morphology, without the confounding influence of surgical or therapeutic intervention. Inclusion criteria were studies: in the English language; involving patients aged ≤ 30; in which cam morphology was measured using radiographical parameters such as the α-angle; and that were specifically designed to compare cohorts with different activity levels without intervening treatments. Intervention studies, even those incidentally reporting preoperative measurements, were excluded to avoid selection bias towards symptomatic populations and overestimation of the association between activity levels and cam and minimise heterogeneity. Additional exclusion criteria included non-original articles, technical notes, editorials, commentaries, conference abstracts, involved surgical or therapeutic interventions that might alter the natural course of cam morphology, or if they were published before 1999, which is the year the term FAI was introduced to the literature [1]. If any disagreements were reached a third author (VK) was consulted.

### Data extraction

Data was extracted from full texts by two authors (M.A., M.P.) and organised in a data charting form that was jointly developed. Data charted was stratified according to activity levels with data for low and high activity groups recorded. For each group data extracted included the number of participants, their age, sex, primary sport, type of imaging used, α-angle used to define cam, training frequency, cam prevalence per hip and individual, and mean α-angles where available.

For data charting, if α-angles were measured by a 3D modality such as MRI or CT, and were reported around a clock-face, values closest to 1:30 was utilised to minimise heterogeneity as they are reported to be most similar to the results of lateral radiographs [17–22]. Where multiple cohorts were available within a study, for instance with prospective follow-up, the most skeletally mature cohort with closed physis was chosen for data extraction. Given the heterogeneity in how studies defined ‘high’ and ‘low’ activity groups (Table I), we retained the original studies’ definitions for our primary analysis as these thresholds were determined by the original authors based on their specific study populations and sport types. An overview of the included studies and the results is shown in Table II.

**Table I:** Definition of high and low activity groups across included studies. (Abbreviations; PAQ-A: Physical activity questionnaire [1–5 scale])

Author (Year)	Sport	High Activity Group Definition	Low Activity Group Definition
Polat et al. (2019)	Football	Training hours: >12.5 h per week Duration of play: ≥ 3 years	Training hours: <12.5 h per week Duration of play: <3 years
Lahner et al. (2014)	Football	Semi-professionals: Minimum of four 2-hour sessions per week for 10-month seasons.	Amateurs: Max 5 h per week
Falotico et al. (2019)	Football	> 3 times per week over 5 years	Non-athlete volunteers
Tak et al. (2015)	Football	≥ 4 times a week since < 12 years old	≤ 3 times a week since < 12 years old
Johnson et al. (2012)	Football	3 times a week, > 36 weeks per year	2 times a week, < 26 weeks per year
Siebenrock et al. (2011)	Basketball	Uninterrupted participation in club training program; 3–8 sessions a week. Each athlete participated in the program since age 8, for 9.2 ± 4.3 years.	Non-athletes: <2 h of sports per week
Westermann et al. (2021)	Various	Power sports athletes; PAQ-A 2.5 ± 0.8 (1–5 scale)	Non-athletes; PAQ-A 2.1 ± 0.8 (1–5 scale)
Abrahamson et al. (2020)	Ski	Active at 5-year follow up: 5.3 ± 1 days per week	Retired at 5-year follow up: 3.8 ± 1.6 days per week
Ayeni et al. (2022)	Various	3.56 ± 2.08 h of sport per week	3.25 ± 2.22 h of sport per week

**Table II:** Overview of results. (Abbreviations; PAQ-A: Physical activity questionnaire, XR: X-Ray, AP: Anteroposterior, DXA: Dual-energy X-ray Absorptiometry, MRI: Magnetic Resonance Imaging, M: Male, F: Female)

Author: Sport	Study type	α-angle criterion	Imaging Modality	Age	Sex (*n* individual) [*n* hip]	Training Frequency	Prevalence of Cam Morphology, %		Mean α-angle	Quality assessment score (%)
							Per individual	Per hip		
**Polat et al. 2019: Football**	Cross-sectional	α-angle > 55°	XR; AP Pelvis and frog lateral							47
Low activity group				10–17	M (135)	Training hours: <12.5 h per week Duration of play: <3 years	By training hours: 22.9% By duration of play: 13.7%	NA	NA	
High activity group				10–17	M (79)	Training hours: >12.5 h per week Duration of play: ≥ 3 years	By training hours: 41.7% By duration of play: 39.5%	NA	NA	
**Lahner et al. 2014: Football**	Cross-sectional	α-angle > 55°	MRI of bilateral hips without contrast							78
Low activity group				22.5 (18–29)	M (22) [44]	Amateurs: Max 5 h per week	27.3%	29.5%	51.7° ± 4.8°	
High activity group				23.3 (18–30)	M (22) [44]	Semi-professionals: Minimum of four 2-hour sessions per week for 10- month seasons	63.6%	47.7%	57.3° ± 8.2°	
**Folatico et al. 2019: Football**	Cross-sectional	α-angle > 82°	Pelvic anteroposterior radiography							63
Low activity group				29.9 (± 5.6)	M (32) [64]	Non-athlete volunteers	28.1%	28.1%	67.5° ± 8.4°	
High activity group				25.1 (± 4.8)	M (60) [120]	> 3 times per week over 5 years	91.6%	92.5%	84.6° ± 6.3°	
**Tak et al. 2015: Football**	Cross-sectional	α-angle > 60°	XR; AP Pelvis and frog lateral							69
Low activity group				23.1 (18.2–38.4)	M (41) [82]	≤ 3 times a week since < 12 years old	NA	42%	NA	
High activity group				23.1 (18.2–38.4)	M (22) [44]	≥ 4 times a week since < 12 years old	NA	64%	NA	
**Johnson et al. 2012: Football**	Cross-sectional	α-angle ≥ 55°	XR; AP Pelvis and frog lateral							44
Low activity group				18–30	M (25) [50] F (25) [50]	2 times a week, < 26 week per year	56% (M) 32% (F) 44% (Total)	39%	55.4° (M) 48.5° (F)	
High activity group				18–30	M (25) [50] F (25) [50]	3 times a week, > 36 weeks per year	60%(M) 36% (F) 48% (Total)	40%	57.5° (M) 50° (F)	
**Siebenrock et al. 2011: Basketball**	Cross-sectional	α-angle ≥ 55°	MRI							81
Low activity group			Aged matched to high activity group		M (38) [76]	Non-athletes: <2 h of sports per week	NA	NA	47.4° ± 4.3	
High activity group			17.6 (9–25)		M (37) [72]	Uninterrupted participation in club training program; 3–8 sessions a week Each athlete participated in the program since age 8, for 9.2 ± 4.3 years	NA	NA	60.5° ± 9.1	
**Westerman 2021: Various**	Prospective cohort	α-angle ≥ 55°	DXA scan L hips only							88
Low activity group				Baseline: 17 Follow up: 23	At baseline: M (40) F (52)	Non-athletes; PAQ-A 2.1 ± 0.8 (1–5 scale)	Baseline: 5.4% 5-year follow up: 5.4%		Baseline: 41.5° ± 7.1° 5-year follow up: 42°	
High activity group				Baseline: 17 Follow up: 23	At baseline: M (55) F (63)	Power sports athletes; PAQ-A 2.5 ± 0.8 (1–5 scale)	Baseline: 6.8% 5-year follow up: 14.4%		Baseline: 43.6° ± 9.5° 5-year follow up: 46°	
**Abrahamson 2020: Ski**	Prospective cohort	α-angle ≥ 55°	Bilateral Hip MRI							95
Low activity group				23.6	M (20) F(24)	Retired at 5-year follow up: 3.8 ± 1.6 days per week	NA	35%	NA	
High activity group				23.6	M (10) F (6)	Active at 5-year follow up: 5.3 ± 1 days per week	NA	43%	NA	
**Ayeni et al. 2022: Various**	Prospective cohort	α-angle ≥ 55°	MRI							87
Low activity group				14–16	M (1) [1] F (3) [3]	3.25 ± 2.22 hours of sport per week	NA	25%	54.18° ± 3.81	
High activity group				14–16	M (20) [20] F (8) [8]	3.56 ± 2.08 h of sport per week	NA	64%	57.64° ± 6.83	

### Study quality assessment

Included articles underwent quality assessment by two authors (M.A., M.P.) using the Downs and Black checklist [23]. Items referring to an intervention were excluded (items 4,8,13,14,15,16,17,19,23,24) and validity was assessed using the remaining items, the results of the completed checklist can be found in Table I and supplementary Table II. The authors discussed ambiguities in the checklist prior to the quality assessment. In case of disagreement the senior author (V.K.) was consulted.

### Data synthesis and presentation

In line with the primary aim of the review, to map the available evidence regarding the association between activity levels and cam, we charted data on (1) prevalence of cam morphology both per hip and per individual and (2) α-angle measurements across high and low activity groups.

For each included study, the odds ratio (OR) was calculated to represent the likelihood of cam in high activity groups compared to low activity groups. The odds ratios were charted separately for studies reporting prevalence per hip and those reporting prevalence per individual. The mean difference in α-angle values across high and low activity groups was also calculated. Figures were generated to visualise odds ratios and the mean α-angle differences between high and low activity groups.

To explore potential relationships and a threshold effect between time spent exercising per week and cam development, we compiled data on training time where reported. For studies that only reported training frequency (e.g., times per week) rather than hours per week, mean weekly training duration was estimated through literature search based on published training regimens of representative sporting groups [24–26]. These estimates allowed plotting mean or estimated hours of training per week against cam prevalence per hip across studies, allowing visualisation of this relationship.

## Results

The literature search yielded 730 studies, of which nine were included for final analyses after screening. These included a total of 890 participants, of which 684 were males and 206 were females. Ayeni et al. (2020) [27], Westermann et al. (2021) [28] and Abrahamson et al. (2020) [29] reported the same cohorts at varying points in prospective follow-up, whereas the remainder of the included studies were cross-sectional. An overview of the results is displayed in Table II.

### Prevalence of cam morphology

#### Prevalence per hip

Six studies reported the prevalence of cam per hip in low and high activity groups [27, 28, 30–33]. Studies used varying imaging modalities such as MRI [27, 30], anteroposterior and frog-lateral radiographs [31–33] and DXA scans [28]. α-angle thresholds for defining cam morphology ranged from > 55° to > 82°. Definitions of high activity within the included studies varied and ranged from 3.56 ± 2.08 h per week [27] to ≥ 4 times weekly since age 12 [32] as summarised in Table I. Study populations included football players [31–33] and mixed sports of football, hockey and basketball [27, 28], with ages ranging from 14 to 30 years.

**Fig. 2:**
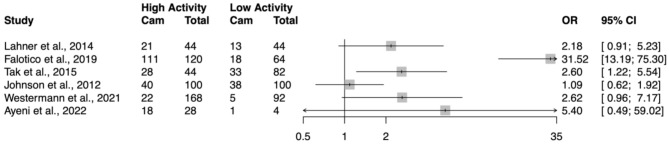
Odds ratios with 95% confidence intervals for cam morphology based on prevalence per hip, comparing high vs. low activity groups across included studies. Odds ratios greater than 1.0 indicate higher likelihood of cam morphology in high activity groups

The OR for cam prevalence per hip between high and low activity groups in each study is shown in Fig. 2. Despite the heterogeneity between studies, all reported a higher prevalence of cam per hip in high activity groups compared to low activity groups, with OR ranging from 1.09 [33] to 31.52 [31] and five of the studies reporting OR > 2. Four studies demonstrated statistical significance [28, 30–32].

#### Prevalence per individual

The prevalence of cam per individual in low and high activity groups was reported in three studies, all examining football players [30, 33, 34] with ages ranging from 10 to 30 years. Imaging modalities included MRI [30] and anteroposterior with frog-lateral radiographs [33, 34]. Cam morphology was defined using an α-angle threshold of > 55° across all studies. Definitions of high activity within the included studies also varied, ranging from four 2-hour sessions weekly [30] to > 12.5 h per week [34] as shown in Table I.

**Fig. 3:**
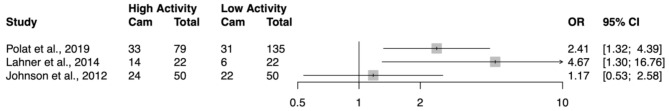
Odds ratios with 95% confidence intervals for cam morphology based on prevalence per individual, comparing high vs. low activity groups across included studies. Odds ratios greater than 1.0 indicate higher likelihood of cam morphology in high activity groups

The OR for cam prevalence per individual between high and low activity groups in each study is shown in Fig. 3. Similarly to prevalence per hip, all studies of the studies reported a higher prevalence of cam per individual in high activity groups with OR ranging from 1.17 [33] to 4.67 [30]. Two studies demonstrated statistical significance [30, 34].

### α-angle

Six studies reported the α-angles in high vs. low activity group [27, 28, 30, 31, 33, 35]. Three of these studies reported α-angles on MRI images [27, 30, 35], with the remaining studies using antero-posterior with or without lateral radiographic views [28, 31, 33]. The studies demonstrate greater α-angle values in the high activity groups.

**Fig. 4:**
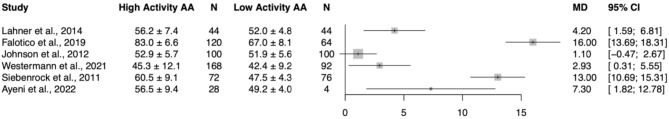
The mean difference in α-angle values (in degrees) between high and low activity groups across included studies, with 95% confidence intervals. Positive values indicate larger α-angles in high activity groups. (Abbreviations; AA: α-angle)

The data from these studies is presented in Fig. 4 to visualise the mean difference in α-angles between high and low activity groups within each study. All six studies reported higher mean α-angle values in high activity groups comparing to low activity groups, with differences ranging from 1.10° [33] to 16.00° [31]. Four studies demonstrated a statistically significant difference [28, 30, 31, 35]. The largest difference in α-angle were observed in studies by Siebenrock et al. (13.00°) and Falotico et al. (16.00°), which examined basketball players and football players, respectively.

### Training dose-response relationship and potential thresholds

**Fig. 5:**
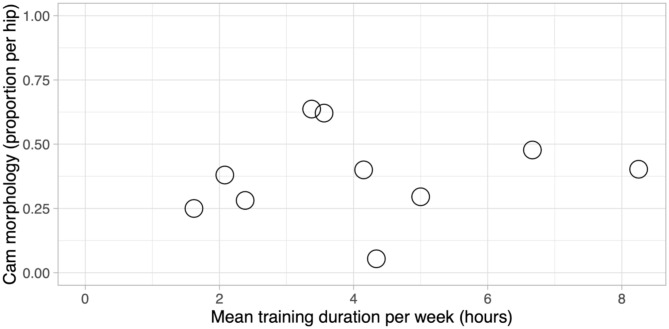
Scatter plot of cam morphology prevalence as a proportion per hip in relation to mean training duration per week. Each circle represents a cohort from the included studies

While all studies compared high vs. low activity, the reporting of specific training parameters in terms of time and frequency varied considerably across studies. Five studies reported cam prevalence as proportion per hip [27, 30–33] and two as proportion per individual [30, 33] with sufficient information available to estimate mean hours of training per week. Figure 5 maps the reported mean hours of training per week in relation to cam prevalence per hip from studies that reported this information. Cam prevalence values ranged from near 0–95% across reported training duration. Notably, there is wide variation in cam prevalence at similar training durations, particularly around 3–4 h per week where the prevalence values ranged from approximate 5–65%.

### Potential confounding factors and heterogeneity across studies

We identified several potential confounding factors which may influence the observed association between activity levels and cam morphology and contribute to increased heterogeneity within studies which preclude valid comparisons to be made. Age ranges varied across studies, from 10 [34] to just over 30 years old [30, 31]. Sex distribution also differed, with five studies including only males [30–32, 34, 35], four studies including both sexes [27–29, 33] and a male predominance overall (77% of participants). The typing of sporting activity also varied, with football being the commonest [30–34] (5 studies), followed by mix sports including hockey and basketball [27, 28] (2 studies), basketball [35] (1 study) and skiing [29] (1 study).

Imaging modalities differed across studies, including MRI [27, 29, 30, 35] (4 studies), antero-posterior and frog-lateral radiographs [31–34] (4 studies) and DXA scans [28] (1 study), with α-angle thresholds for defining cam morphology ranging from > 55° to > 82°. Hip dominance was reported in three studies [27, 30, 31]. These variations in study populations and methodological approaches represent important considerations when interpreting the mapped evidence.

### Quality assessment

The methodological quality of the included studies ranged from 44 to 95%, with higher scores indicating better quality (supplementary materials – Table II). Cross-sectional studies scored between 44 and 81% whereas prospective cohort studies demonstrated higher quality scoring 87–95%. All studies performed well in reporting quality (items 1–3, 6–7), with consistently clear descriptions of objectives, outcomes and main findings. External validity (items 11–12) was generally poor, with most studies lacking information about population representativeness. Studies performed well on statistical testing and outcome measurement accuracy (items 18, 20) but demonstrated weakness in selection bias (items 21–22, 25–26). Few studies conducted a power calculation (item 27).

## Discussion

The purpose of our study was to map the available evidence on the association between activity levels and cam morphology and elucidate the nature of this association and whether a threshold effect exists at specific training intensities or durations. Our results suggest that the prevalence of cam per hip and per individual is greater in high activity groups across the included studies. Similarly, the α-angle values appear generally greater in high activity groups.

The mapping of the literature indicates an association between high sporting activity and cam morphology, although the precise nature of this relationship remains unclear due to significant heterogeneity in study designs, definitions of activity levels and measurement methods of α-angles. This heterogeneity presents challenges to drawing definitive conclusions about the relationship between specific training parameters and cam development, and whether a threshold effect might exist.

### Cam prevalence and α-angle values

The illustrative figures presenting cam prevalence per hip (Fig. 2) and per individual (Fig. 3) suggest a potential trend of higher cam prevalence in high activity groups across the included studies. This observation aligns with literature exploring the effect of sporting exposure on hip morphology [7, 8] and supports the notion of an adaptive aetiology for cam development whereby increased stress on the hip joint and repetitive micro-trauma may predispose to the condition [32].

The variation in reported prevalence across studies likely reflects the different criteria used to stratify individuals into high and low activity groups, in addition to other factors such as the type of sport, imaging modalities and baseline characteristics of the cohorts including sex and age at which sporting exposure began. Male-only cohorts appeared to show potentially stronger associations between activity levels and cam morphology compared to mix-sex cohorts. This may suggest that sporting activity has a greater influence on cam development in males. The authors postulate, if this is the case, that it may be due to the delayed age of physeal closure in males and increased exposure to sporting activity both in aggregate activity, and higher intensity training regimens older adolescent athletes take part in [36].

Similar to the findings on cam prevalence, illustrative presentation of α-angle values (Fig. 4) suggests larger α-angle values in high activity groups compared to low activity groups. This observation provides additional support for a relationship between sporting activity and cam development, although the same limitations regarding heterogeneity across studies apply.

### Nature of the association between activity levels and cam

The evidence mapped in this review suggests an important association between athletic activity and cam morphology, which could possibly be a dominant risk factor influencing the development of cam in young adults. Therefore, in accordance with the second aim of this review, we aimed to identify the nature of any relationship between activity levels in terms of hours of training per week and cam. Tak et al. (2015) [32] previously demonstrated a significant dose-response relationship between training frequency and cam in adolescent soccer players.

Figure 5 maps the available data on cam prevalence per hip in relation to reported or estimated mean hours of training per week. The data points exhibit considerable variability, particularly at similar training durations around 3–4 h per week. This heterogeneity highlights a significant gap in the literature regarding the specific relationship between training volume and cam development. The paucity and lack of standardised reporting of exercise parameters represent a challenge in elucidating the nature between activity levels and cam. Few studies reported detailed information on training intensity, frequency, and duration or distinguished between types of athletic loads.

Furthermore, where training parameters were reported, definitions of high vs. low activity groups and measurement methods of α-angles varied substantially, complicating direct comparisons across studies and the ability to draw definitive conclusions. Our findings suggest that while there appears to be an association between high sporting activity levels and cam morphology development, the specific parameters of exercise time and frequency that might constitute ‘safe’ training regimens remain unclear. This collectively highlights the need for detailed and standardised reporting of activity levels within the literature. In order to properly characterise the relationship between activity levels and cam development across sporting activities, prospective studies are required where adolescents are clearly stratified by activity levels with explicit data collection and reporting. This would enable the generation of evidence-based guidelines for adolescent sporting activity to reduce the risk of cam development.

While our review focuses on exercise time and frequency as metrics for activity levels, the type of sporting activity and specific movement patterns may be equally or more influential in cam development. Impingement, cutting and contact sports are associated with greater prevalence of cam compared to endurance, flexibility and asymmetric sports [8]. Additionally, positional differences within sports (e.g. goalkeepers vs. outfield players in ice hockey) have also been shown to influence cam morphology development [7, 8]. These findings suggest that specific biomechanical loads and movement patterns, rather than simply duration of activity may be critical in the pathogenesis of cam morphology. The relationship between exercise parameters in terms of time and frequency and cam development should therefore be interpreted with the context of sport-specific biomechanical demands.

### Future directions

Based on the knowledge gaps identified in this scoping review, several areas for future research emerge. First, prospective longitudinal studies following adolescents through skeletal maturity with standardised reporting of activity levels and imaging protocols are needed to establish temporal relationships between activity levels and cam development. Second, studies should employ consistent and detailed reporting of activity parameters that consider frequency, duration and intensity to allow for investigation of potential dose-response relationships and facilitate more meaningful comparisons across studies. Third, investigation of specific movement patterns and biomechanical loads rather than simply duration and frequency of activity may provide more nuanced understanding of the mechanisms underpinning cam development. Finally, research exploring potential threshold effects or ‘safe’ training parameters could inform evidence-based guidelines for youth sports participation.

### Strengths & limitations

This study represents the first scoping review to comprehensively map the evidence on the association between activity levels and cam morphology, including both exercise frequency and duration. Despite a broad literature search across multiple databases and varied resources, there are however several limitations. The primary limitation was the significant heterogeneity across included studies, with inconsistent definitions of high vs. low activity groups, varying sport types, participant demographics and imaging modalities complicating direct comparisons. The substantial variation in activity level classifications, ranging from specific hours per week to frequency of training sessions, limited our ability to characterise potential dose-response relationships or identify threshold effects. Furthermore, by including only English-language publications, we may have missed relevant studies in other languages.

Our methodology did not allow for quality assessment of evidence or establishment of causality, and the exclusion of intervention studies, while intentional to focus on natural relationships, may have omitted controlled study data. We also identified limited research examining the influence of activity levels on cam morphology while controlling for potential confounding factors such as genetic predisposition, physical maturity variation, body composition and differences between training intensity vs. duration which may influence the observed association. Finally, our decision to chart data according to original studies’ definitions of activity levels, while necessary given the variation, limits direct comparability of findings across different sporting contexts.

## Conclusion

This scoping review maps the current evidence suggesting that cohorts reporting high sporting activity levels (approximately 3–8 training sessions per week or 3.5–12.5 h of weekly training) have a greater prevalence of cam morphology and larger α-angle values vs. low activity cohorts. However, significant knowledge gaps exist in understanding the specific relationship between exercise time and frequency and cam development. The heterogeneity in study designs, definitions of activity levels and reporting of exercise parameters limits our ability to characterise specific dose-response relationships or identify a threshold effect. Prospective cohort studies with standardised reporting of activity levels and clear stratification of adolescent sporting activity are needed to better characterise this relationship and inform evidence-based guidelines for youth sports participation.

## Electronic supplementary material

Below is the link to the electronic supplementary material.

Supplementary Material 1

## Data Availability

Data is provided within the manuscript or supplementary information files.

## Abbreviations

FAIS: Femoroacetabular impingement syndrome
PRISMA: ScR-Preferred Reporting Items for Systematic Reviews and Meta-Analyses extension for Scoping Reviews
MRI: Magnetic Resonance Imaging
CT: Computed Tomography
3D: Three Dimensional
DXA: Dual-energy X-ray Absorptiometry
XR: X-ray
AP: Anteroposterior
CI: Confidence Interval
PAQ: A-Physical Activity Questionnaire
M: Male
F: Female
OR: Odds Ratio
AA: α-angle
