# Supplementary material for: The effect of exercise time and frequency on the development of cam morphology
Source: BMC Musculoskelet Disord. 2025 Oct 1;26:903. doi: 10.1186/s12891-025-08603-1 (PMC12486972; doi:10.1186/s12891-025-08603-1)
Supplement: Supplementary file 1 — Supplementary Material 1 [file 12891_2025_8603_MOESM1_ESM.pdf]

Supplementary materials - Table I. Search strategy

Embase			Medline			Cochrane Library		
**Query**		**Results**	**Query**		**Results**	**Query**		**Results**
1.	exp time factor/	46,113	1.	exp time factor/	777,442	1.	MeSH descriptor: [Time Factors] explode all trees	72994
2.	Time.ab,kw,ti.	4,638,945	2.	Time.ab,kw,ti.	2,636,498	2.	(Time):ti,ab,kw	478790
3.	Frequency.ab,kw,ti.	1,083,310	3.	Frequency.ab,kw,ti.	626,940	3.	(Frequency):ti,ab,kw	92954
4.	Activity level.ab,kw,ti.	19,260	4.	Activity level.ab,kw,ti.	11,354	4.	(Activity level):ti,ab,kw	39051
5.	Duration.ab,kw,ti.	953,455	5.	Duration.ab,kw,ti.	481,337	5.	(Duration):ti,ab,kw	186923
6.	(hours per week or hours per day).ab,kw,ti.	8,956	6.	(hours per week or hours per day).ab,kw,ti.	4,814	6.	((hours per week or hours per day or activity level)):ti,ab,kw	51860
7.	(training adj3 (frequen* or intens*)).ab,kw,ti.	19,968	7.	(training adj3 (frequen* or intens*)).ab,kw,ti.	12,082	7.	(training NEAR/3 (frequen* or intens*)):ti,ab,kw	8626
8.	(intens* adj3 (exercise or sport* or activ*)).ab,kw,ti.	46,082	8.	(intens* adj3 (exercise or sport* or activ*)).ab,kw,ti.	30,162	8.	(intens* NEAR/3 (exercise or sport* or activ*)):ti,ab,kw	13300
9.	(amount adj3 (exercise or sport* or activ*)).ab,kw,ti.	9,077	9.	(amount adj3 (exercise or sport* or activ*)).ab,kw,ti.	5,746	9.	(amount NEAR/3 (exercise or sport* or activit*)):ti,ab,kw	827
10.	exp etiology/ or aetiology.ab,kw,ti. or etiology.ab,kw,ti. or etiology.fs.	3,457,618	10.	exp etiology/ or aetiology.ab,kw,ti. or etiology.ab,kw,ti. or etiology.fs.	1,907,805	10.	(aetiology or etiology):ti,ab,kw	102133
11.	exp prevalence/ or prevalence.ab,kw,ti.	1,288,482	11.	exp prevalence/ or prevalence.ab,kw,ti.	682,797	11.	MeSH descriptor: [Prevalence] explode all trees	8680
12.	exp Risk/ or exp Risk Factors/ or risk*.ab,kw,ti.	4,686,548	12.	exp Risk/ or exp Risk Factors/ or risk*.ab,kw,ti.	2,657,343	12.	("prevalence"):ti,ab,kw	43271
13.	exp femoroacetabular impingement/	4,755	13.	exp femoroacetabular impingement/	2,419	13.	MeSH descriptor: [Risk] explode all trees	58210
14.	FAI.ab,kw,ti.	4,441	14.	FAI.ab,kw,ti.	2,467	14.	MeSH descriptor: [Risk Factors] explode all trees	33011
15.	(femoroacetabular impingement* or femoro-acetabular impingement*).ti,ab,kw.	4,551	15.	(femoroacetabular impingement* or femoro-acetabular impingement*).ti,ab,kw.	2,717	15.	(Risk*):ti,ab,kw	289382

16.	Cam deformity.ab,kw,ti.	296	16.	Cam deformity.ab,kw,ti.	179	16.	MeSH descriptor: [Femoracetabular Impingement] explode all trees	115
17.	Cam-type.ab,kw,ti.	687	17.	Cam-type.ab,kw,ti.	394	17.	(FAI):ti,ab,kw	429
18.	Cam morphology.ab,kw,ti.	254	18.	Cam morphology.ab,kw,ti.	135	18.	(femoroacetabular impingement* or femoro-acetabular impingement*):ti,ab,kw	195
19.	1 or 2 or 3 or 4 or 5 or 6 or 7 or 8 or 9	6,158,568	19.	1 or 2 or 3 or 4 or 5 or 6 or 7 or 8 or 9	3,942,802	19.	(cam deformity):ti,ab,kw	26
20.	10 or 11 or 12	8,076,083	20.	10 or 11 or 12	4,367,506	20.	(cam morphology):ti,ab,kw	31
21.	13 or 14 or 15 or 16 or 17 or 18	7,826	21.	13 or 14 or 15 or 16 or 17 or 18	4,255	21.	(cam-type):ti,ab,kw	17
22.	19 and 20 and 21	598	22.	19 and 20 and 21		22.	#1OR#2OR#3OR#4OR#5OR#6OR#7OR#8OR#9	673952
						23.	#23 #10OR#11OR#12OR#13OR#14OR#15	391537
						24.	#24 #16OR#17OR#18OR#19OR#20OR#21	577
						25.	#25 #22AND#23AND#24	53

Supplementary materials - Table II. Quality assessment of included studies performed using the Downs and Black checklist.

Author, year	*Q1*	*Q2*	*Q3*	*Q4*	*Q5*	*Q6*	*Q7*	*Q8*	*Q9*	*Q10*	*Q11*	*Q12*	*Q13*	*Q14*	*Q15*	*Q16*	*Q17*	*Q18*	*Q19*	*Q20*	*Q21*	*Q22*	*Q23*	*Q24*	*Q25*	*Q26*	*Q27*	*Total*	*%*
Cross-sectional studies																													
Siebenrock et al., 2011	1	1	1	n/a	2	1	1	n/a	n/a	1	1	0	n/a	n/a	n/a	n/a	n/a	1	n/a	1	0	0	n/a	n/a	1	n/a	1	13	81
Johnson et al., 2012	1	1	1	n/a	0	1	0	n/a	n/a	1	0	0	n/a	n/a	n/a	n/a	n/a	1	n/a	1	0	0	n/a	n/a	0	n/a	0	7	44
Tak et al., 2015	1	1	1	n/a	0	1	1	n/a	n/a	1	0	0	n/a	n/a	n/a	n/a	n/a	1	n/a	1	1	1	n/a	n/a	1	n/a	0	11	69
Polat et al., 2019	1	1	1	n/a	0	1	1	n/a	n/a	0	0	0	n/a	n/a	n/a	n/a	n/a	1	n/a	1	n/a	n/a	n/a	n/a	0	n/a	0	7	47
Falotico et al., 2019	1	1	1	n/a	1	1	1	n/a	n/a	1	0	0	n/a	n/a	n/a	n/a	n/a	1	n/a	1	0	0	n/a	n/a	0	n/a	1	10	63
Lahner et al. 2014	1	1	1	n/a	2	1	1	n/a	n/a	1	0	0	n/a	n/a	1	n/a	1	1	n/a	1	0	0	n/a	n/a	1	n/a	1	14	78
Prospective cohort studies																													
Westermann et al. 2021	1	1	1	n/a	2	1	1	n/a	n/a	1	1	0	n/a	n/a	0	n/a	n/a	1	n/a	1	1	0	n/a	n/a	1	1	1	15	88
Abarahmson et al. 2020	1	1	1	n/a	1	1	1	n/a	1	1	1	1	n/a	n/a	n/a	n/a	1	1	n/a	1	1	1	n/a	n/a	1	1	1	18	95
Ayeni et al. 2022	1	1	1	n/a	2	1	1	n/a	1	n/a	1	0	n/a	n/a	1	n/a	n/a	1	n/a	1	1	0	n/a	n/a	n/a	1	n/a	13	87
